# Employment, Education, and Income for Canadians with Developmental Disability: Analysis from the 2017 Canadian Survey on Disability

**DOI:** 10.1007/s10803-020-04603-3

**Published:** 2020-07-13

**Authors:** Patrick Berrigan, Craig W. M. Scott, Jennifer D. Zwicker

**Affiliations:** 1 The School of Public Policy, University of Calgary, 906 - 8 Ave SW 5th floor, Calgary, AB T2P 1H9 Canada; 2 Faculty of Kinesiology, University of Calgary, 376 Collegiate Blvd NW, Calgary, AB T2N 4V8 Canada

**Keywords:** Canadian survey on disability, Employment, Education, Income, Developmental disability

## Abstract

This study assessed needs and outcomes for people with developmental disability (DD) to understand the socioeconomic status of this group prior to implementation of the Accessible Canada Act in June 2019. The 2017 Canadian Survey on Disability (CSD) was used to analyze data for a sample of individuals with self-reported disability. Data related to employment, education, income, housing, caregivers, and use of government benefits is reported. Compared to the general Canadian public, persons with DD are less likely to: finish high-school or post-secondary education; participate in the labor force or be employed; and earn on average less/year in total income. To align with recent accessibility legislation, significant progress is needed to address disparities for people with DD.

## Introduction

The Accessible Canada Act aims to eliminate barriers and to ensure greater opportunities for people with disability in Canada (Employment and Social Development Canada 2019). Specifically, the act sets out to create a culture change, through monitoring and oversight, with respect to access for people with disabilities in areas under federal jurisdiction including buildings/public spaces, employment opportunities, information/communication technologies, delivering public programs/services, and transportation (Employment and Social Development Canada 2019).

Developmental disability (DD) is a common type of disability, defined as an impairment in cognitive function that presents prior to adulthood and persists throughout a person’s life (Government of Ontario 2016). The number of individuals impacted by DD in Canada is large. Estimates of the percentage of children in Canada with DD have ranged from 6.5 to 8.3% and many people with DD experience lifelong limitations that impact their quality of life (Arim et al. 2017; Lamsal et al. 2018; Zwicker et al. 2017). Despite its substantial impact, there is a lack of up-to-date information on accessibility and inclusion outcomes for people with DD in Canada. Previous analysis has shown that Canadians with disability face barriers to employment and education resulting in poorer standard of living outcomes on average than the general public (Zwicker et al. 2017). However, these findings are based on data collected in 2012 by the Canadian Survey on Disability (CSD) and it is unclear if they reflect the current reality for people with DD in Canada (Statistics Canada 2014).

A subsequent version of the CSD was conducted in 2017, representing an opportunity to update the literature on outcomes for people with DD. We therefore report data on employment, education, income, housing, caregivers, and use of government benefits for people with DD using the 2017 CSD. An understanding of the current state of economic and standard of living outcomes for Canadians with DD is critical to develop targeted policies to improve the lives of people with DD and also for monitoring Canada’s progress towards meeting the goals of the Accessible Canada Act.

For context, in Canada supports for people with disability are provided by the federal, provincial, and municipal governments and also by not-for-profit organizations (Mccoll et al. 2017). Programs that provide housing and housing supports for people with disability exist across all branches of government in Canada (Heart and Stroke Foundation of Canada 2020). Additionally, not-for-profits play an important role in assisting people with disabilities in obtaining housing. With respect to education, the federal government mandates that children must receive education (though not necessarily public education) and that public education must be accessible to all children (Statistics Canada 2008). Based on these guidelines, each province is left to implement their own system.

## Methods

### The Canadian Survey on Disability

The CSD is a national repeated cross-sectional survey of Canadians 15 years of age and above who are limited because of a health-related problem or condition (Cloutier et al. 2018). CSD participants were recruited from individuals who reported having a long-term condition or difficulty on the 2016 Census Long-Form Questionnaire. From this sample, people with disability were identified using the Disability Screening Questions (DSQ). The DSQ are questions developed by Statistics Canada and Employment and Social Development Canada, to identify people with disability from larger samples (Grondin 2012).

The 2017 CSD classified disability into 10 types consisting of hearing, vision, mobility, flexibility, dexterity, pain, learning, mental health, memory, and developmental disabilities. To facilitate further classification, CSD respondents were asked to report the medical conditions that cause them the most difficulty or limitations to their daily activities, to a maximum of two. Statistics Canada recoded the reported conditions with the corresponding International Classification of Diseases, Tenth Revision (ICD-10) code (WHO 2010). This allowed for the identification of subgroups based on diagnostic classification beyond the 10 types of disability pre-specified by the 2017 CSD.

Data collected by the CSD focused on four broad themes disability characteristics, supports/barriers, education, and employment (Cloutier et al. 2018). Additionally, Statistics Canada has linked respondents’ 2017 CSD data with their 2016 Census data, allowing for a greater range of variables. Statistics Canada has also linked 2016 Census data for a sample of the non-disabled population to facilitate comparisons between groups with disability and non-disabled people (Cloutier et al. 2018).

Vetting requirements imposed by Statistics Canada stipulate that data for samples of less than 10 individuals cannot be released and are replaced with an “X” in this analysis. Furthermore, data must meet minimum reliability thresholds. Data with a coefficient of variation (CV) between 16.6 and 33.3% is marked with an “E”, indicating that readers should use caution regarding the value. Data with a CV of over 33.3% cannot be reported and is replaced with an “F”. Of note, the 2017 CSD user guide recommends the CV be calculated by dividing the standard error (SE) of an estimate by the estimate itself.

The 2017 CSD was conducted between March 1 and August 31, 2017 and was administered using an internet-based electronic questionnaire. The 2017 CSD had a 69.5% response rate corresponding to approximately 50,000 participants (Cloutier et al. 2018). Additional information related to the development and administration of the 2017 CSD can be found in the survey’s user guide (Cloutier et al. 2018).

### Sample Groups

This study focuses on people with DD and provides subgroup analysis of two DD, autism spectrum disorder (ASD) and cerebral palsy (CP). ASD is characterized by impairments to social interaction/communication and restricted/repetitive behaviors (Centers for Disease Control and Prevention 2019; Dudley et al. 2015; Filipek et al. 1999; Nicholas et al. 2018). CP is characterized by mental and physical impairments caused by prenatal or early life brain injury (Krigger 2006). Outcomes for people with DD are compared to people who reported having any disability (AD) and non-disabled people for context.

The DD group reflects 2017 CSD respondents who reported that they had been diagnosed with a developmental disability/disorder. For AD, respondents were limited to those who reported that their disability was likely to be lifelong or of unknown duration, as barriers facing people with non-transient disabilities are likely to require different policy solutions than people with transient disabilities. The ASD group included all those who reported either a primary or secondary condition that was linked to an ASD ICD-10 code. ASD codes included F84.0, F84.1, and F84.5. The CP group reflects those who reported either a primary or secondary condition that was linked to a CP ICD-10 code. CP codes included G80.0, G80.1, G80.2, G80.3, G80.4, G80.8, and G80.9.

### Statistical Analysis

Data in the present study is reported using descriptive statistics consisting of means or proportions and their respective 95% confidence intervals (CI). Additionally, in some cases counts are reported. Estimates of means, proportions, and counts were weighted at the individual level to reflect the Canadian population. Since CSD respondents represent a sample of the larger Canadian population with disability, Statistics Canada provides a set of 1000 bootstrapped weights unique to each respondent to account for sample variability. Means, proportions, and counts presented in the present study reflect an aggregation of these 1000 respondent specific weights. Analysis was undertaken using Stata Version 16 with the survey analysis package.

Statistics Canada recommends that comparisons for significance of data from the 2017 CSD be made using CI calculated with bootstrapped SE. To adhere to this recommendation, the present study provides CI for all means and proportions based on bootstrapped SE. 95% CI were calculated by multiplying the bootstrapped SE rounded to one decimal place by 1.96 and using this value to create the interval. Significance can be assessed by whether or not the intervals of two means or proportions overlap.

As there exist known links between age and sex with employment, income, and standard of living outcomes and since the distribution of these variables is not equivalent between included groups (See Table 1), we conducted exploratory analysis controlling for these variables using ordinary least squares (OLS) and logistic regression (Blau and Kahn 2017). Outcomes for which age and sex were controlled for include post-secondary education completion rates, employment income, and home-maintainer status.

**Table 1:** Demographics information, Canada, age > 15, 2017

Category of disability	Average age (CI)	% Female (CI)	% Rural (CI)
Any disability	55.4 (55.2–55.6)	55.7% (54.9–56.5%)	18.5% (17.7–19.3%)
Developmental Disability	38.5 (36.9–40.1)	39.5% (35.0–44.0%)	18.4% (15.1–21.7%)
Autism Spectrum Disorder	28.1 (24.3–32.0)	31.7% (20.5–42.9%) (E)	14.2% (9.3–19.1%) (E)
Cerebral Palsy	37.7 (31.6–43.8)	61.2% (47.1–75.3%)	7.1% (3.2–11.0%) (E)
Non-Disabled	44.3^a^	49.9% (49.7–50.1%)	17.9% (17.5–18.3%)

All values have been rounded to one decimal place

The average age for non-disabled people was taken from the 2016 census

E use with caution, CV (16.6–33.3%)

^a^The standard error rounded to 0 resulting in no range for the CI

## Results

### Demographic Information

The average age of people with DD is 38.5 years old, six years younger than the average of those without disability and 17 years younger than the average of people with AD (Table 1). The average age of people with ASD and CP are 28.1 and 37.7 years old, respectively. While approximately half of non-disabled people in Canada are female and 55.7% of people with AD are female, among people with DD more are male (60.5%). Notably, the female to male ratio for ASD is approximately 1:2 while for CP it is approximately 2:1. Geographically, the percentage of people with DD who live rurally (18.4%) is similar to people with AD (18.5%) or no disability (17.9%). However, there is a geographic difference in location for specific diagnoses. Most strikingly, only 7.1% of people with CP live in rural areas. The CSD defined rural as an area with a population of less than 1,000 or a population density of less than 400 per square kilometer.

Table 2 shows the estimated number of people by both category of disability and age demographic in Canada. People with DD made up 5.2% of the 5,677,170 people over the age of 15 with disability. The percentage of the Canadian population over 15 with DD was 1.0% and for AD it was 18.5%, for ASD it was 0.2%, and for CP it was 0.1% (Statistics Canada 2020b).

**Table 2:** Number of people with disability, Canada, by age, 2017

Category of disability	15 to 24	25 to 54	55 to 64	65 +	Total 15 +
Any disability	417,230 (9.3%)	2,093,980 (14.0%)	1,225,090 (24.1%)	1,940,880 (31.6%)	5,677,170 (18.5%)
Developmental disability	87,790 (2.0%)	143,000 (1.0%)	40,450 (0.8%)	26,500 (0.4%)	297,750 (1.0%)
Autism spectrum disorder	30,840 (0.7%)	27,360 (0.1%)			58,200 (0.2%)
Cerebral palsy	4060 (0.1%)	12,370 (0.0%)^a^			16,430 (0.1%)

All values rounded to the 10

Age specific counts for autism spectrum disorder and CP respectively were combined to meet vetting requirements

^a^Zero percent is the result of rounding

Table 3 shows the estimated percentage of provincial populations by category of disability. Interestingly, the rate of DD appears to be lowest in the Territories Region consisting of the Northwest Territories, Nunavut, and the Yukon. These are Northern regions partially located within the arctic.

**Table 3:** Percentage of provincial population with disability, by province, age > 15, 2017

Province	Any disability	Developmental disability	Autism spectrum disorder	Cerebral palsy
Alberta	17.9%	0.9%	0.1%	0.1%
British Columbia	20.0%	1.1%	0.2%	0.1%
Manitoba	19.9%	1.0%	0.2%	0.1%
New Brunswick	22.8%	1.2%	0.2%	0.1%
Newfoundland & Labrador	20.7%	1.0%	0.2%	0.1%
Nova Scotia	25.9%	1.1%	0.2%	0.1%
Ontario	20.2%	1.1%	0.2%	0.1%
Prince Edward Island	21.6%	0.9%	(X)	(X)
Quebec	13.4%	0.8%	0.1%	(X)
Saskatchewan	19.1%	0.9%	0.1%	0.1%
Territories Region	16.1%	0.5%	(X)	(X)

Data has been rounded to one decimal place

X Suppressed to meet vetting requirements

The Northwest Territories, Nunavut, and The Yukon were merged to meet vetting requirements

### Education Outcomes

**Fig. 1:**
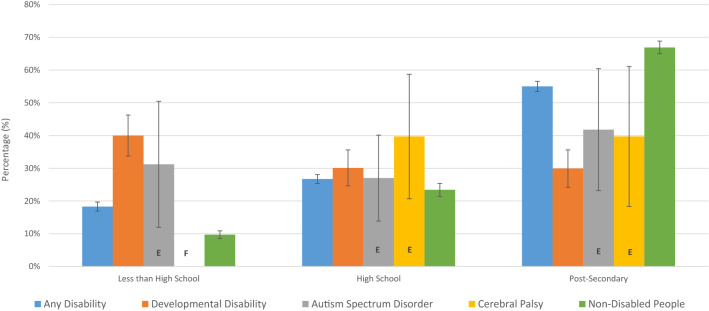
Highest level of education attained, Canada, age 25–64, 2017

People with DD were less likely to complete high school or post-secondary education than non-disabled people and people with AD. Figure 1 shows the estimated highest level of education attained by category of disability. Of note, respondents can only be in one category in Fig. 1. For example, if a respondent had both a high school diploma and a university degree, they would be counted in post-secondary but not high school. The percentage of people not completing high school was 40.0% for DD, 18.3% for AD, and 9.7% for non-disabled people. The percentage of people with less than post-secondary was 70.1% for DD, 45.0% for AD, and 33.1% for non-disabled people. Educational attainment was similar for people with ASD and CP.

As age and sex are likely to influence if a respondent has completed post-secondary education and these variables are not evenly distributed between people with disability and non-disabled people, these variables were controlled for using logistic regression (Van Hek et al. 2016). We found odds ratios (OR) for DD of 0.195 (SE = 0.028), for AD of 0.630 (SE = 0.022), for ASD of 0.300 (SE = 0.128), and for CP of 0.250 (SE = 0.121). As these ORs are less than one, they suggest that the probability of completing post-secondary education is lower for people with disability than for non-disabled people, controlling for age and sex. Each of these ORs were significant at a 1% level.

Educational supports are important for ensuring accessibility of education. We report educational supports required by people with disability and if supports were received. Table 4 shows the estimated percentage of individuals who reported having access to a required educational support for the five most commonly required by category of disability. The most commonly required supports for people with DD were (i) individualized education plans, (ii) extended test time, (iii) modified curriculum, (iv) technology, and (v) a teacher’s aide/tutor. There is substantial similarity amongst the most commonly required supports among categories of disability. For AD, DD, and ASD the top five most commonly required educational supports are the same, though their ordering differs. CP differed from the other categories of disability in that people with CP required supports for physical barriers such as accessible classrooms or specialized transportation.

**Table 4:** Most required supports, Canada, age > 15, 2017

Any disability		Developmental disability		Autism spectrum disorder		Cerebral palsy	
Reported need		Reported need		Reported need		Reported need	
1. Extended test time	87%	1. IEP	80%	1. IEP	84%	1. Technology	86%
2. Technology	68%	2. Extended test time	90%	2. Extended test time	93%	2. Extended test time	100%
3. IEP	81%	3. Modified curriculum	82%	3. Teacher’s aide/tutor	77%	3. Accessible classroom	96%
4. Modified curriculum	71%	4. Technology	68%	4. Modified curriculum	86%	4. Specialized transport	92%
5. Teacher’s aide/tutor	71%	5. Teacher’s aide/tutor	77%	5. Technology	75%	5. IEP	88%

All values rounded to the nearest percent

IEP individualized education plan

Technology includes: Cell/smart phone with specialized features; Computer/table with special software/adaptions; Recording equipment or note taking devices; Device for playing audiobooks; Textbooks in e-format; Magnifiers; Closed-circuit devices; Large print reading materials; Braille reading materials or manual brailler

Table 5 shows the estimated percentage of individuals who reported having access to a required educational support for the five least commonly provided by category of disability. The five least commonly provided supports for people with DD were (i) accessible residences, (ii) speech therapists, (iii) accessible buildings, (iv) technology, and (v) teacher’s aide/tutor. Of note, there is a degree of overlap between the supports reported in Tables 4 and 5. This indicates that some of the most commonly required supports by people with disability are amongst the least commonly provided. ASD and CP were not included in Table 5 because of suppressed data due to high variability.

**Table 5:** Least commonly provided supports, Canada, age > 15, 2017

Any disability		Developmental disability	
Reported need		Reported need	
1. Accessible residences	0%	1. Accessible residences	63%
2. Attendant care services	50%	2. Speech therapist	64%
3. Speech therapist	60%	3. Accessible buildings	67%
4. Accessible buildings	67%	4. Technology	68%
5. Technology	68%	5. Teacher’s aide/tutor	77%

All values rounded to the nearest percent

Technology: see notes for Fig. 5

### Employment Outcomes

**Fig. 2:**
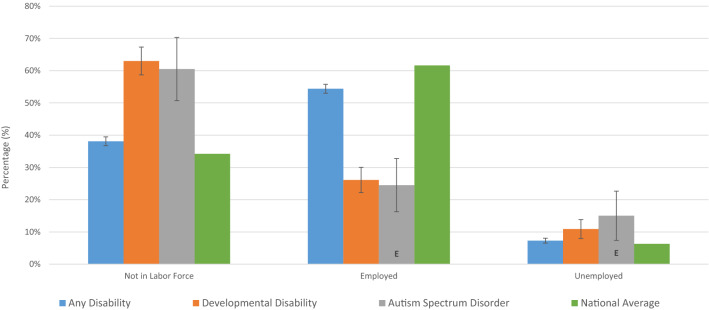
Employment statistics, Canada, age 15–64, 2017

Figure 2 shows the estimated labor market outcomes by category of disability. For DD, 63.0% of people were not in the labor force, 10.9% were unemployed, and 26.1% were employed. In contrast, the national averages indicate 34.2% were not in the labor force, 6.3% were unemployed, and 61.6% were employed. People with DD also fared worse when compared to people with AD, having lower labor force participation (37.0% versus 61.9%) and employment (26.1% versus 54.4%). Of people with disability who were employed, fewer people with DD worked full-time, as 51.9% (CI 42.9–60.9%) of DD were full time, 78.3% (CI 76.7–79.9%) of AD were full time, 44.9% (CI = 26.1%-63.7% E) of ASD were full time, and 39.3% (CI 15.6–63.0% E) of CP were full time. Values for national averages are from Statistics Canada and reflect people 15 and over (Statistics Canada 2019e). CP was not included in Fig. 2 because of suppressed data due to small sample size.

The 2017 CSD, asked individuals not in the labor force what barriers prevented them from working. Table 6 shows the top five reasons given by category of disability. The same five barriers were identified for both the DD and AD groups (i) health condition, (ii) lack of training, (iii) no jobs available, (iv) past attempts unsuccessful, and (v) a fear of losing current supports. Though the barriers reported were the same between AD and DD, the percentage of individuals reporting a given barrier was higher for DD. ASD and CP were not included in Table 6 because of suppressed data due to both small sample size and high variability.

**Table 6:** Barriers to employment, Canada, Age 15–64, 2017

Any disability		Developmental disability	
Barrier		Barrier	
1. Health condition	10.1% (9.3–10.9%)	1. Health condition	20.5% (16.4–24.6%)
2. Lack of training	7.8% (7.0–8.6%)	2. Lack of training	13.9% (10.8–17.0%)
3. No jobs available	7.1% (6.3–7.9%)	3. Past attempts unsuccessful	13.1% (9.6–16.6%)
4. Past attempts unsuccessful	6.4% (5.6–7.2%)	4. No jobs available	11.9% (8.8–15.0%)
5. Lose current supports^a^	4.4% (3.8–5.0%)	5. Lose current supports	9.9% (6.2–13.6%) (E)

All values rounded to one decimal place

E use with caution, CV (16.6–33.3%)

^a^Was tied with experienced Discrimination

The 2017 CSD collected data on the workplace accommodations required by people with disability and if respondents received the accommodation. Table 7 shows the estimated percentage of individuals who reported having access to a required workplace accommodation for the five most commonly required by category of disability. For DD, the most commonly required accommodations are (i) modified duties, (ii) modified work hours, (iii) human support, (iv) working from home, and (v) special computer software. CP was not included in Table 7 because of suppressed data due to both small sample size and high variability.

**Table 7:** Most required accommodations, Canada, age > 15, 2017

Any disability		Developmental disability		Autism spectrum disorder	
Reported need		Reported need		Reported need	
1. Modified work hours	48%	1. Modified duties	32%	1. Modified duties	28%
2. Modified duties	38%	2. Modified work hours	35%	2. Modified work hours	15%
3. Chair with back support	38%	3. Human support	30%	3. Human support	16%
4. Working from home	34%	4. Working from home	10%	4. Working from home	(X)
5. Modified workstation	41%	5. Special computer software	20%	5. Special computer software	11%

All values rounded to the nearest percent

X Suppressed to meet vetting requirements

Table 8 shows the estimated percentage of individuals who reported having access to a required workplace accommodation for the five least commonly met accommodations by category of disability. For DD, the least commonly met accommodations were (i) working from home, (ii) chair with back support, (iii) modified workstation, (iv) accessible building features, and (v) special computer software. There was little similarity between the least commonly met accommodations between DD and AD. Additionally, rates at which accommodations were met tended to be lower for DD than AD. Similar to supports for education there was a degree of overlap between the accommodations reported in Tables 7 and 8, suggesting that some of the most commonly required workplace accommodations are the least provided. ASD and CP were not included in Table 8 because of suppressed data due to both small sample size and high variability.

**Table 8:** Least commonly met accommodations, Canada, Age > 15, 2017

Any disability		Developmental disability	
Reported accommodation		**Reported accommodation**	
1. Specialized transportation	17%	1. Working from Home	10%
2. Technical aids	24%	2. Chair with back support	10%
3. Special computer software	26%	3. Modified workstation	13%
4. Communication aids	29%	4. Accessible building features	16%
5. Accessible elevator	29%	5. Special computer software	20%

All values rounded to the nearest percent

Table 9 highlights the top five industries of employment for people with disability using the North American Industry Classification System (NAICS) by category of disability (Statistics Canada, 2019d). For DD, the most common industries of employment are (i) retail trade, (ii) healthcare & social assistance, (iii) accommodations & food services; (iv) construction, and (v) manufacturing. ASD and CP were not included in Table 9 because of suppressed data due to both small sample size and high variability.

**Table 9:** Employment by industry, Canada, age 15—64

Any disability		Developmental disability	
Industry		Industry	
1. Retail trade	9.2% (8.4–10.0%)	1. Retail trade	7.9% (5.7–10.1%)
2. Healthcare & social assistance	8.7% (7.9–9.5%)	2. Healthcare & Social Assistance	4.1% (2.5–5.7%) (E)
3. Manufacturing	5.6% (4.8–6.4%)	3. Accommodations & Food Services	4.0% (2.6–5.4%) (E)
4. Construction	5.0% (4.4–5.6%)	4. Construction	3.9% (1.9–5.9%) (E)
5. Professional, scientific, & technical services^a^	4.7% (3.9–5.5%)	5. Manufacturing	2.8% (1.2–4.4%) (E)

All values rounded to one decimal place

E Use with caution, CV (16.6–33.3%)

^a^Professional Services was tied with Educational Services and Accommodations & Food Services

### Income and Government Transfers

**Fig. 3:**
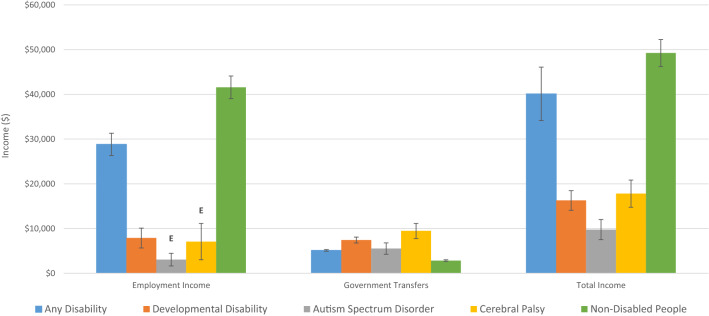
Average before tax income, Canada, age 15–64, 2015

Income is critical to ensuring full participation in society. We report estimated before tax employment income, government transfers, and total income by category of disability (Fig. 3). People with DD earned less on average than non-disabled people or people with AD. This discrepancy exists for both employment and total income. The discrepancy was particularly striking for the ASD group. Though people with DD receive more government transfers ($7446 versus $2820), their total income on average is still only one third of non-disabled people ($16,283 versus $49,235). When comparing DD to AD, government transfers were $7446 for DD versus $5116 for AD but total income for people with DD was only 40.6% of people with AD ($16,283 versus $40,106). People with CP received more government transfers than people with ASD ($9467 versus $5533) and earned more in total income ($17,815 versus $9765).

The poorer labor market outcomes and inadequate government transfers has meant that many people with DD qualify as low-income. When measured using the Market Basket Measure (MBM) 28.2% (CI 23.7–32.7%) of people with DD, 17.0% (16.2–17.8%) of people with AD, 24.1% (CI 12.3–35.9% E) of people with ASD, and 37.6% (CI 21.5–53.7% E) of people with CP are considered low-income. This compared to the 10.5% (CI 10.3–10.7%) of non-disabled people who earn less than the MBM threshold. The MBM measures low-income based on a household’s ability to purchase a basket of commonly used goods that corresponds to an acceptable standard of living (Statistics Canada 2019c). This does not account for additional costs associated with disability that people without disability would not incur.

As age and sex are likely to influence employment income and these variables are not evenly distributed between people with disability and non-disabled people, we controlled for these variables to see if they contribute to the discrepancy (Van Hek et al. 2016). Using OLS we found differences of − $31,191 (SE = $$1358) for DD, − $16,091 (SE = $1328) for AD, − $30,220 (SE = $1521) for ASD, and − $30,013 (SE = $3555) for CP. As these differences are negative, they suggest that employment income is lower for people with disability than for non-disabled people, controlling for age and sex. Each of these differences were significant at a 1% level.

### Housing

**Fig. 4:**
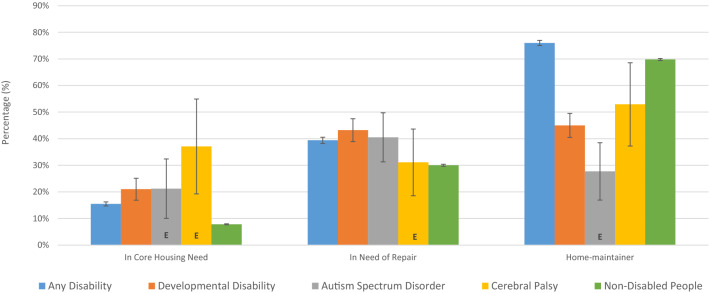
Housing outcomes, Canada, age > 15, 2017

Figure 4 shows housing outcomes including the estimated percentage of individuals living in core-housing need, the estimated percentage of individuals living in housing in need of repair, and the estimated percentage of individuals who are household maintainers by category of disability. Briefly, core-housing need refers to housing that fails to meet standards for adequacy, suitability, and affordability and a household maintainer refers to someone who is entirely or in part responsible for household payments (Statistics Canada, 2019a, b). People with DD were more likely than non-disabled people to be in core housing need (21.0% versus 7.8%), more likely to be in housing in need of repair (43.2% versus 30.0%), and less likely to be a home maintainer (45.0% versus 69.8%). Compared to AD, people with DD were more likely to be in core housing need (21.0% versus 15.5%) and less likely to be a home-maintainer (45.0% versus 76.0%). Results were similar between people with ASD and CP.

Counterintuitively, data from the 2017 CSD suggests that people with AD are more likely to be home-maintainers than non-disabled people. We hypothesized that this discrepancy may be driven by age and sex differences between these groups. We base this hypothesis on the fact that age and sex can impact income and a barrier to housing for persons with disability in Canada is a lack of affordability. Consequently, we controlled for these variables. Using logistic regression, we found OR for DD of 0.392 (SE = 0.040), for AD of 0.924 (SE = 0.029), ASD 0.291 (SE = 0.077) and for CP of 0.669 (SE = 0.203). As these ORs are less than one, they suggest that the probability of being a home-maintainer is lower for people with disability than for non-disabled people, controlling for the impact of age and sex. Each of these ORs were significant at a level of at least 2% with exception of CP, which did not meet standard thresholds for significance. After controlling for age and sex, people with AD were less likely to be home-maintainers than non-disabled people, in line with expectations.

### Supports for People with Disability

**Fig. 5:**
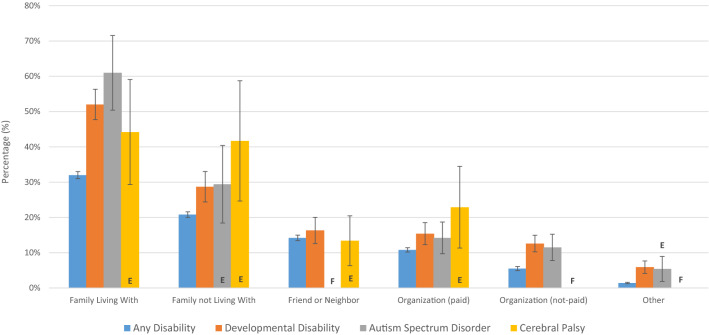
Caregivers for people with disability, Canada, age > 15, 2017

Figure 5 shows estimates of who provides care for people with disability by category of disability. For DD, 52.0% of caregivers were family members the individual was living with, 28.7% were family members the individual was not living with, 16.3% were friends or neighbors, 15.4% was a paid organization, and 12.6% was an unpaid organization. For all categories of disability, the most common caregivers were family members.

**Fig. 6:**
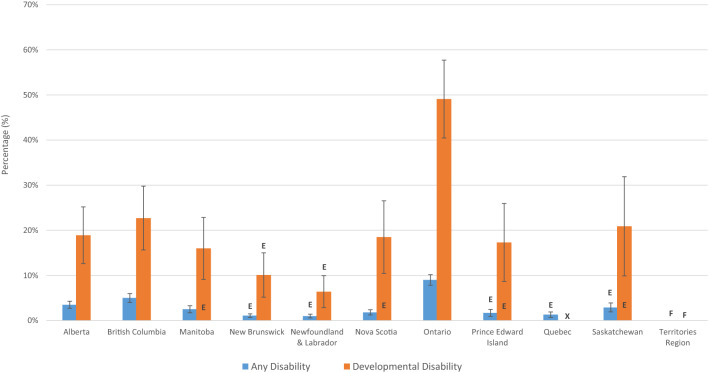
Provincial benefit receiptients, by province, age > 15, 2017

Benefit programs targeted at people with disability are one of the main ways in which governments attempt to address disparities for groups with disability. Figure 6 shows the estimated percentage of people with DD and AD who report being the beneficiary of a provincial disability support program by category of disability. For DD, the lowest percentage of people receiving provincial benefits was in Newfoundland and Labrador at 6.4% and the highest was in Ontario at 49.1%. For all provinces, the percentage of people with DD receiving provincial benefits was higher than that for AD. ASD and CP were not included in Fig. 6 because of suppressed data due to both small sample size and high variability.

In addition to provincial benefits, the federal government offers the Registered Disability Savings Plan (RDSP) (Government of Canada 2019). This program serves as a tax deduction for people with disability. However, uptake of this benefit is minimal at approximately 0.2% for people with AD. DD, ASD, and CP were not reported because of suppressed data due to both small sample size and high variability.

## Discussion

*We are impacted by the inability to secure our son’s future. We are his sole social circle, we are his financial backers, we are his transportation — we are his life. My fears keep me awake at night. If we don’t have something in place — a plan, a program, a support network — what will happen to my son when I’m gone? Institutionalized, neglected, or worse, homeless, with no love or supports — I need peace of mind and he needs a future.* – Parent statement for the Canadian Senate Standing Committee 2018. The aforementioned quote serves as a testament to the realities faced by people with DD and their families. Situations such as this are a reminder as to the need for inclusivity and provide context for the outcomes presented in this study.

Our findings suggests that people with DD in Canada experience lower educational attainment, have poorer labor market outcomes, and have poorer housing situations than non-disabled people. Compared to the general Canadian public, people with DD are 4.1 times less likely to finish high school; 2.1 times less likely to finish post-secondary education; 1.8 times less likely to participate in the labor force; 2.4 times less likely to be employed; 2.7 times more likely to live in inadequate housing, and earn on average $32,952 less/year in total income. These findings are largely in line with analyses of the 2012 CSD (Zwicker et al. 2017). However, direct temporal comparisons cannot be made between surveys, as the process used to identify respondents differed (Cloutier et al. 2018).

With respect to the demographics of people with disability, the younger average age of people with ASD identified in the present study is consistent with a recent trend of increased ASD diagnoses. Estimates by the Center for Disease Control suggest that autism prevalence increased approximately 2.5 times between 2000 and 2014 in the United States (Centers for Disease Control and Prevention 2019). Furthermore, higher rates of ASD diagnoses in males is in line with known trends in ASD (Government of Canada 2018). With respect to geography, only 7.1% of people with CP live in rural areas, possibly indicating a move to an urban center given the need for an accessibly built environment. The lower rates of disability in northern regions identified in the present study is possibly explained by a lack of diagnostic capacity. Research suggests that people living in northern regions lack access to healthcare (Huot et al. 2019). This lack of access may be resulting in less diagnostic capacity for DD and as a result fewer diagnoses.

Education is crucial to improved career prospects and life outcomes regardless of disability status. However, compared to the general Canadian public, our findings suggest that people with DD are 4.1 times less likely to finish high school and 2.1 times less likely to finish post-secondary education. This is consistent with findings in the United States suggesting that people with intellectual disabilities (ID) have the lowest rate of postsecondary enrollment of any group with disability at 28.7% and enrolment drops to just 6.7% when considering four year college programs (Newman et al. 2011). An important distinction regarding high school completion rates in groups with disability is between those who dropout because of a lack of supports versus those who age out without meeting the academic standards for a high school diploma. Individuals who age out without meeting the necessary academic standards, often receive a certificate of completion which is not equivalent to a high school diploma. Previous research in Canada has suggested that 18.2% of children with severe or very severe disability will dropout prior to completing high-school (Statistics Canada 2013).

Low rates of postsecondary completion could be a potential driver of the comparatively poor labor force outcomes for people with DD. Our findings suggest that people with DD are 1.8 times less likely to participate in the labor force, 2.4 times less likely to be employed, and earn on average $32,952 less/year in total income, relative to non-disabled people. This is unsurprising in light of previous research suggesting that postsecondary education increases the odds of successful employment for people with DD and increases the likelihood of working more hours and earning higher wages (Cimera et al. 2018; Grigal et al. 2011).

Income disparities for people with DD are striking and our findings suggest that government benefit programs are not adequately meeting the needs of people with DD. This is most prominently demonstrated by the difference in total income between people with DD and non-disabled people. Non-disabled people earn on average three times that of people with DD. Further compounding this issue is the inability of people with DD to access benefits and services. Only 10.1–49.1% of those with DD surveyed were beneficiaries of provincial benefit programs for people with disability depending on province and only 0.2% of people with AD were using the RDSP. This low uptake may suggest that programs are not appropriately targeted to people with disability (Dunn and Zwicker 2018). Either being overly burdensome and complicated or administered through the tax system. Many people with DD do not file taxes.

The cumulative effect of low income and inadequate access to benefit programs has likely contributed to the high number of people with DD living in poverty, 28.2% when measured by the MBM. This is likely an understatement, as the MBM does adequately account for costs incurred by people with disability specific to their condition that non-disabled people would not have to pay. People with DD were also more likely to live in inadequate housing (21.0% versus 7.8%) and more likely to live in housing in need of repair (43.2% versus 30.0%) compared to non-disabled people. Improving access to programs, benefits, and services represents an essential step in improving outcomes for people with DD.

The industries that employed people with DD are similar to those for AD and the general Canadian public. Based on Statistics Canada data, the top industries of employment in Canada for 2017 by number of employees were: (i) Healthcare and Social Assistance; (ii) Retail Trade; (iii) Manufacturing; (iv) Professional, Scientific, & Technical Services; and (v) Construction (Statistics Canada 2019d). It is important to point out that there are differences in ordering in the industries of employment between people with DD and the general Canadian Population. For example, Accommodations & Food Services is the third most common industry of employment for people with DD but only the seventh most common for the general Canadian public.

Our findings suggest that family members are the most common caregivers for adults with DD. These findings potentially speak to the degree to which unpaid caregiving occurs in Canada (Lilly et al. 2010). It has been estimated that there are 7.8 million Canadians who care for either a family member or friend with disability (Statistics Canada 2020a). This caregiving, which tends to be disproportionately done by women, likely has a substantial impact on Canada’s economy, as previous research suggests that caregiving affects the labor market decisions and productivity of caregivers (Lilly et al. 2010).

The barriers reported in the CSD can provide guidance on areas for policymakers to focus their efforts in addressing disparities. Commonly reported barriers to employment include a fear of losing disability supports, a lack of appropriate job training, and a lack of success during previous attempts to find employment. Based on these barriers, programs that incentivize companies to hire people with DD, less aggressive claw backs of benefits for people with DD who enter the workforce, and additional training programs for people with DD could represent targeted policy approaches to improve outcomes. It is important to point out that policy efforts cannot be made in the absence of people with disability. For disability related policy to be effective in achieving its objective, it is important that people with disability be central in the policy-making process.

In almost all of the comparative analysis presented in the present study that included people with CP, individuals with CP had comparatively worse outcomes than the general Canadian population or people with AD. These findings are largely in line with existing research on employment, education, and standard of living outcomes for people with CP (Zwicker et al. 2017). Previous research has suggested that individuals with CP are subject to a compounding effect whereby lower educational attainment translates into lower employment outcomes and income (Huang et al. 2013; Vogtle 2013). Huang et al. (2013) suggest targeted educational and vocational supports are required for people with CP. These supports are particularly important during times of transition, such as when youth first enter the workforce or begin independent living.

There are limitations associated with data collected from the 2017 CSD. The CSD does not capture data for people: younger than 15; living on first nations reserves; living in collective dwellings such as senior facilities, assisted living facilities, correctional facilities, hospitals, military bases; or for the homeless. As a result, the CSD likely missed a substantial portion of people with disability. Furthermore, beyond the 10 pre-specified disability types, respondents needed to specify additional conditions to be categorized in these groups. As a result, the survey likely missed a portion of people with ASD and CP who did not report these disabilities. Given the limitations of the 2017 CSD, we speculate that the sample represents a relatively high functioning portion of Canadians with disability. Given this survey is administered as an online questionnaire, there are potential sampling selection and representability issues (Evans and Mathur 2005). To mitigate these, Statistics Canada identified participants who would be less likely to access the online survey and contacted these individuals if they did not respond to the 2017 CSD to offer alternative interview formats (Cloutier et al. 2018).

This study describes the status of people with DD in Canada prior to the introduction of accessibility legislation, focusing on employment, education, income, housing, caregivers, and use of government benefits. Findings indicate that there exists substantial disparity between people with DD and the general Canadian public and a need for reducing barriers in communities, schools, and workplaces. To close these gaps legislation guaranteeing equal participation at the provincial level for people with disability will be beneficial. Though federal laws exist, only Manitoba, Nova Scotia and Ontario currently have accessibility legislation. Furthermore, merely adopting these policies is not sufficient. It is important that these laws are enforced. Secondly, barriers to accessing supports need to be removed wherever possible. For example, using tax credits, as a mechanism to support people with disability may not be a useful approach. Finally, workplace and educational accommodations need to be made available for people who need them. This study found that some of the most commonly required work and educational accommodations/supports were the least commonly provided.
